# Incentivizing Monitoring and Compliance in Trophy Hunting

**DOI:** 10.1111/cobi.12120

**Published:** 2014-01-01

**Authors:** NILS BUNNEFELD, CHARLES T T EDWARDS, ANAGAW ATICKEM, FETENE HAILU, E J MILNER-GULLAND

**Affiliations:** * Department of Life Sciences, Imperial College LondonSilwood Park, Ascot, SL5 7PY, United Kingdom; † School of Natural Sciences, University of StirlingStirling, FK9 4LA, United Kingdom; ‡ Centre for Ecological and Evolutionary Synthesis (CEES), Department of Biology, University of OsloP.O. Box 1066, Blindern, NO-0316, Oslo, Norway; § Ethiopian Wildlife Conservation Authority (EWCA)P.O. Box 386, Addis Ababa, Ethiopia

**Keywords:** adaptive management, conflict, harvesting, natural resources, social-ecological system, socioeconomics, sustainability, colecta, conflicto, manejo adaptativo, recursos naturales, sistema socio-ecológico, socioeconomía, sustentabilidad

## Abstract

*Conservation scientists are increasingly focusing on the drivers of human behavior and on the implications of various sources of uncertainty for management decision making. Trophy hunting has been suggested as a conservation tool because it gives economic value to wildlife, but recent examples show that overharvesting is a substantial problem and that data limitations are rife. We use a case study of trophy hunting of an endangered antelope, the mountain nyala (Tragelaphus buxtoni), to explore how uncertainties generated by population monitoring and poaching interact with decision making by 2 key stakeholders: the safari companies and the government. We built a management strategy evaluation model that encompasses the population dynamics of mountain nyala, a monitoring model, and a company decision making model. We investigated scenarios of investment into antipoaching and monitoring by governments and safari companies. Harvest strategy was robust to the uncertainty in the population estimates obtained from monitoring, but poaching had a much stronger effect on quota and sustainability. Hence, reducing poaching is in the interests of companies wishing to increase the profitability of their enterprises, for example by engaging community members as game scouts. There is a threshold level of uncertainty in the population estimates beyond which the year-to-year variation in the trophy quota prevented planning by the safari companies. This suggests a role for government in ensuring that a baseline level of population monitoring is carried out such that this level is not exceeded. Our results illustrate the importance of considering the incentives of multiple stakeholders when designing frameworks for resource use and when designing management frameworks to address the particular sources of uncertainty that affect system sustainability most heavily*.

Incentivando el Monitoreo y el Cumplimiento en la Caza de Trofeos

**Resumen:**

*Científicos conservacionistas cada vez se enfocan más en los conductores del comportamiento humano y en las implicaciones de varias fuentes de incertidumbre en la toma de decisiones de manejo. La cacería de trofeos ha sido sugerida como una herramienta de conservación porque le otorga valor económico a la vida silvestre, pero ejemplos recientes muestran que la sobrecolecta es un problema sustancial y que las limitaciones de datos son abundantes. Usamos el estudio de caso de la cacería de trofeos de un antílope en peligro, el nyala de las montañas (Tragelaphus buxtoni), para explorar como las incertidumbres generadas por el monitoreo de poblaciones y el tráfico de especies interactúan con la toma de decisiones de 2 actores clave: las compañías de safari y el gobierno. Construimos un modelo de evaluación de estrategia de manejo que incluye la dinámica poblacional del nyala de las montañas, un modelo de monitoreo y un modelo de toma de decisiones de una compañía. Investigamos escenarios de inversión en el combate del tráfico de especies y el monitoreo por el gobierno y las compañías de safari. La estrategia de colecta fue robusta hacia la incertidumbre en los estimados de población obtenidos del monitoreo, pero el tráfico de especies tuvo un efecto más fuerte sobre la cuota y la sustentabilidad; por esto, reducir el tráfico de especies está dentro de los intereses de compañías que desean incrementar la rentabilidad de las empresas, por ejemplo al hacer participar a los miembros de la comunidad como guías de caza. Hay un nivel umbral de incertidumbre en los estimados de población más allá del cual la variación anual en la cuota de trofeos previene la planeación por parte de las compañías de safari. Esto sugiere un papel para el gobierno asegurando que un nivel base de monitoreo de población se lleve a cabo para que este nivel no sea excedido. Nuestros resultados ilustran la importancia de considerar los incentivos de partes interesadas múltiples al designar marcos de trabajo para el uso de recursos y que al designar marcos de trabajo de manejo dirigirse a las fuentes particulares de incertidumbre que afecten más al sistema de sustentabilidad*.

## Introduction

Trophy hunting has been advocated as a tool for biodiversity conservation because it provides monetary incentives for conservation. Three main arguments have been put forward in support of this stance on trophy hunting: it provides economic incentives for sustainable offtake (Lindsey et al. 2007); it turns land into conservation areas, for example over 1.3 million km^2^ in sub-Saharan Africa is used for trophy hunting, which is more than state land provided for wildlife (Lindsey et al. 2006); and it can finance reintroductions, for example black wildebeest (*Connochaetus gnou*) and southern white rhino *(Ceratotherium simum simum*) in South Africa (Leader-Williams et al. 2005). However, a recent study by Packer et al. (2011) showed that intensity of trophy hunting is a significant factor in the decline of hunted populations in Tanzania, indicating that poorly regulated hunting practices can lead to trophy hunting having a negative effect.

Results of a modeling study indicate trophy hunting may be sustainable for some antelope species in some areas of Africa (Caro et al. 2009), but the same study also points out the main challenges of trophy hunting as a conservation tool: limited or no time series of population estimates and widely differing monitoring methods that differ in precision and spatial extent (e.g., aerial survey, foot census). Monitoring efforts must be sufficient to detect relevant rates of change in the population size within a meaningful period or they risk wasting valuable resources (Hockley et al. 2005).

Individual resource user behavior has recently been identified as one of the key uncertainties facing terrestrial conservation (Keane et al. 2008) and fisheries (Fulton et al. 2011) because compliance with rules depends strongly on economic incentives and social and cultural drivers. However, the extent of illegal offtake is hard to quantify; thus, estimates are likely to be highly inaccurate (but see St John et al. 2012 for novel methods to quantify illegal behavior). For example, at the western edges of the Serengeti, illegal bushmeat hunting is estimated to be pursued by 8–57% of households, depending on the study cited (Loibooki et al. 2002; Kaltenborn et al. 2005). The extent of illegal offtake in trophy-hunting systems is rarely considered, but illegal hunting (whether for trophies or by local users for meat) is important to quantify if sustainability of the system is to be maintained (Hilborn et al. 2006).

When managers grant extraction rights to a relatively small number of external users (e.g., safari companies, fishing fleets), conflict is likely because local resource users face restrictions on resource use, whereas others are able to access these same resources. The challenges of conflicts between different resource users have been well documented in fisheries; illegal catch is widespread (Agnew et al. 2009). Fisheries provide a good example of how multiple stakeholders and their varied objectives increase the complexity of management and make agreement over and compliance with rules difficult (Smith et al. 2008). For example, conservation stakeholders may prioritize the conservation of the wider ecosystem, fisheries managers the sustainable management of the harvestable stock, commercial fishers the economic performance of the fleet, and recreational fishers their satisfaction with their fishing experience. Thus traditional strategies and models optimizing against the objectives of a single stakeholder are unlikely to produce consensus on management approaches.

The management strategy evaluation (MSE) framework was developed first for fisheries and has been widely and successfully used (Butterworth & Punt 1999). The main strength of the MSE framework is its ability to test management decisions against various uncertainties, including the population size of the stock and the level of illegal offtake, by modeling the resource dynamics, monitoring process, management decision making, and implementation within a unified framework (Butterworth & Punt 1999; Sainsbury et al. 2000). In fisheries, the application of the MSE framework has also in many cases led to a better commitment to the agreed procedure by all stakeholders because of its transparency and ability to demonstrate the effects of assumptions about all components of a management system (Smith et al. 2008). Recently, there have been proposals to extend the framework to include more realistic trade-offs between socioeconomic and ecological sustainability through the explicit modeling of resource-user decision making (Little et al. 2009; Milner-Gulland et al. 2010; Bunnefeld et al. 2011; Milner-Gulland 2011) but this has not been tested for a terrestrial system.

The mountain nyala antelope (Tragelaphus buxtoni) is an ideal case study for exploring the role of trophy hunting in combining conservation and economic interests in a developing country. It is listed as endangered on the International Union for Conservation of Nature Red List and is endemic to Ethiopia in Conservation International’s Eastern Afromontane biodiversity hotspot (Mittermeier et al. 2005). The species has been hunted for trophies since 1968 (Safari Club International, personal communication). Trophy-hunting data exist since 1996 and suggest an average revenue of US$2,251,000 over 15 years to the Ethiopian Wildlife Conservation Sector (Federal & Regional Governments). The mountain nyala’s current population size is estimated to be just under 4000 individuals, and the population has come under increasing pressure from legal trophy hunting, poaching, and habitat loss (Atickem et al. 2011). Mountain nyala density is 3.7 times higher in areas patrolled by game scouts than in unpatrolled areas. These areas include the Bale Mountains National Park and some controlled hunting areas, where hunting companies invest in game scouts (Atickem et al. 2011). This indicates illegal livestock grazing and direct poaching by local people have a negative effect on mountain nyala densities. The Ethiopian Wildlife Conservation Authority (EWCA) conducts yearly monitoring to assess the nyala population size (a single visit every 2 years to each hunting area), upon which it bases its quota allocation. The allocation has been 14–38 nyala from 1999 to 2009. The EWCA also collects a trophy fee from individual hunters. The safari companies receive a daily fee from hunters, and EWCA sets a minimum safari length of 21 days. A small number of local people are employed as game scouts for the hunting concession areas and support the logistics of the hunting trips.

In most trophy-hunting situations such as for the mountain nyala, there are 3 key stakeholders involved, government, safari companies, and local people. We focused on the decision making of the government and accounted for incentives of safari companies and local people through different scenarios. The government strives to maximize the quota (fees) and keep the risk of extirpation low. The companies aim to maximize the quota and minimize variability of quotas to plan ahead and attract safari hunters. Safari companies are aware that they face uncertain but potentially high losses through illegal offtake. Thus, investment in activities reducing local poaching (either law enforcement or positive incentives for local communities) may indirectly increase quotas in the future as population size increases. We focused on income from trophy-hunting activities because most tourism in Ethiopia is connected to its historical and cultural richness, such as ancient churches. Ecotourism is largely undeveloped, especially in the Bale Mountains, due to a lack of tourism infrastructure, planning, and marketing (Admasu et al. 2011).

Traditional models of harvesting in terrestrial ecology and conservation often assume that the quota is set as a constant proportion of the estimated population size (Lande et al. 1995), whereas fisheries scientists have developed adaptive harvesting rules that allow the quota to be adjusted depending, for example, on previous quotas and population trends (Rademeyer et al. 2007). We sought to compare different strategies of quota setting (constant, proportional, and adaptive harvesting rules) the government can use to model offtake of a trophy-hunted species under uncertainty. We built model scenarios to explore the roles of monitoring (to reduce uncertainty in observed population size) and the level of poaching on the ability to make informed decisions and in determining system performance. Performance is evaluated relative to population size, quota size, and year-to-year variation in quota size, 3 measurements of paramount importance to government and safari companies. A robust approach to setting a quota for species that are of very high conservation and economic value, but are difficult in terms of data availability and management capacity, could lead to major improvements in the potential of trophy hunting as a conservation tool.

## Methods

### Management Strategy Evaluation Submodels

**Figure 1:**
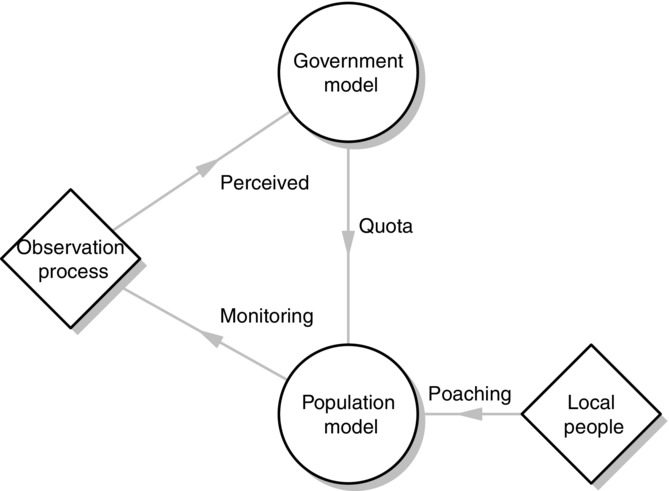
Diagram of the management strategy evaluation framework for trophy hunting of nyala. The population model produces the true population of mountain nyala, the observation model mimics the observation process, and the government makes the decision on the basis of perceived population size. The government then passes the quota on to the safari companies who comply with the quota given by the government. Poaching and the uncertainty in the observation process are represented as diamonds because they are part of the scenario analysis rather than being internal to the model structure.

The MSE modeling framework consists of 3 submodels. (1) A model of mountain nyala population dynamics (operating model) produces the “true” population dynamics. (2) The observation model relates the precision of monitoring to the effort put into monitoring by the government and the safari companies. (3) The government model sets the quota through implementation of a harvest-control rule that is based on the observed population size (Fig.1). Poaching occurs as an external driver in the operating model, but the government is unaware of the poaching rate when setting the quota. All simulations were run for 50 years to keep the length realistic for management and 1000 iterations to account for stochasticity in the model. The last 10 years of each simulation and iteration were used as output.

The operating model was a stage-structured 2-sex matrix model, in which population dynamics was updated following annual monitoring. The model consisted of juveniles (0–1 years), yearlings (1–2 years), and adults (2 to >5 years): 
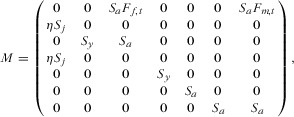
1where *S_j_, S_y_*, and *S_a_* are survival rates of juveniles, yearlings, and adults, respectively (Table1), *F_f,t_* is the fecundity of adult females in year *t*, and *F_m,t_* is the fecundity for male nyala ≥5 years. In the absence of information on nyala vital rates, we parameterized the model with vital rates from a study on greater kudu (*T. strepsiceros*), an ungulate of similar size (Owen-Smith 1990) and general ungulate life-history parameters (Gaillard et al. 1998). We followed Caswell and Weeks (1986) to calculate the relative contribution of male and female fecundity in polygynous mating systems (Supporting Information). We assumed an equal sex ratio after birth (η = 0.5). Density dependence was included in the model by varying juvenile survival (Supporting Information).

**Table 1:** Mountain nyala population dynamics modeled with a matrix model with parameters (SD) from the literature

Parameter	Symbol	Parameter	Source
Survival yearling	*S_y_*	0.849	Owen-Smith 1990
Survival adult	*S_a_*	0.933	Owen-Smith 1990
Litter size	*K*	0.944	Gaillard et al. 2000
Survival yearling SD		0.101	Owen-Smith 1990
Survival adult SD		0.061	Owen-Smith 1990
Fecundity SD		0.065	Gaillard et al. 2000

Given the Population Vector **N**

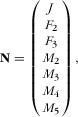
2where *J* is juveniles, *F* is females of 2 and 3 years old, and *M* is males 2–5 years old, the transition between 1 year and the next was calculated by multiplying the matrix (Eq. 1) by the population vector

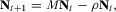
3where ρ is proportion of population poached. The population size was reduced each year by ρ. Poaching rates ranged from 0% to 12% and targeted all stage classes, unlike trophy hunting which targeted only adult (old) males.

The model was run for different degrees of observation uncertainty. Coefficient of variation (CV) ranged from 1% to 20%, which is similar to CVs reported in the literature for large African mammals (Plumptre 2000). We assumed a baseline CV of 15% for monitoring reflected the relatively low investment in monitoring in this case study. We used the CV to calculate the observed population size from a normal distribution around the actual population size **N**, a practice that is consistent with sampling theory.

The government sets the number of males hunted for trophies. The quota is restricted to old males with a horn size of 74 cm, which is thought to be reached at 5 years of age (Evangelista et al. 2007). On the basis of discussions with companies and EWCA, we assumed quotas were implemented fully and without error. The government does not know the poaching levels, and there is high monitoring uncertainty; thus, the harvest-control rule is based on highly uncertain and biased data.

### Trophy Harvesting

We considered 3 types of trophy harvesting: constant, proportional, and adaptive. For constant trophy hunting, a constant number of mountain nyala were harvested every year regardless of population size. Currently 30 nyala are taken per year over the whole area, which suggests this scenario is relatively realistic under current management (EWCA, personal communication). The model was applied to a hypothetical hunting concession that holds about one-third of the total nyala population. Thus, the constant harvest quota in our MSE evaluation was 10 nyala/year taken from the class of males >5 years old.

In the case of proportional harvesting, the number of harvested males >5 years old was determined by multiplying the observed population size by the percent harvest rate and subtracting these individuals from the number of males >5 years old. We tested harvest rates from 0–5%; in the absence of illegal poaching and observation error the population reached extinction at a harvest rate of >4.5% (Supporting Information). We used the proportional harvest rate of 1.2% as the base case because this equaled 10 nyala (old males) at the starting population.

We used the adaptive harvest-control rule to calculate the number harvested (*H*) in year *t*:

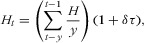
4where the mean harvest over **y** years (sum of the nyala harvest divided by the number of years over which the harvest is considered) is multiplied by the degree to which the harvest is allowed to change (δ) in response to the change in observed population size (τ). The change in population size *τ* was found by fitting a linear model through the observed population size over the number of observation years (*y*) with maximum-likelihood techniques. We call δ the flexibility parameter. For δ = 1, the harvest changed at the same rate as the population, whereas for δ = 0.5 the harvest changed at half that rate. This harvest-control rule was adapted from the fisheries literature (Rademeyer et al. 2008). Different values for *y* and δ were explored (see below for more detail on scenarios). Each simulation started with the current harvest of 10 males >5 years old. In fisheries this approach is called an empirical harvest-control rule because it is based directly on empirical data (past data of population estimates and offtake) to set the quota. The second option often used in fisheries is a model-based harvest-control rule that implements a simulation model to predict future population changes and makes decisions on the basis of these predictions. We used an empirical harvest-control rule because they are more reliable in data-poor situations when model fitting is problematic. They require lower levels of computer power, allowing a more comprehensive range of uncertainties to be explored, and there is a higher probability of stakeholder buy in because they are more likely to understand the decision making process (Rademeyer et al. 2007).

### Performance Metrics

The performance metrics were annual quota, average annual variation (AAV) in the quota, population size, percentage of times the population went extinct out of 1000 replicates, and the CV of population size. We determined these metrics over 10 years at the end of the simulation. The AAV was calculated as (Rademeyer et al. 2008):

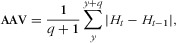
5where *H* is the harvest and *q* the time span of years over which the average was taken.

### Scenarios

**Figure 2:**
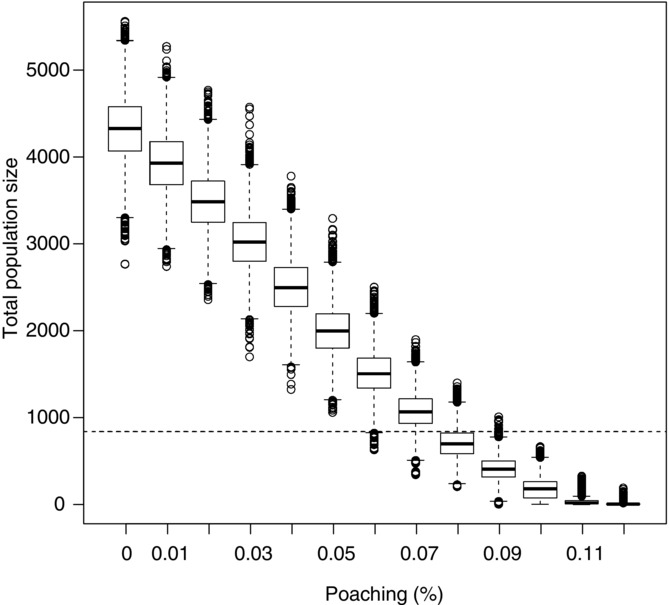
Mean population size for a range of levels of poaching of nyala (percent offtake of the population) for 1000 iterations over 10 years after deleting 40 years of results (horizontal lines, starting population on the basis of current estimated population size).

We first looked at the effect of different poaching levels (1–10% of the population) to evaluate the most likely current poaching rate given the life history of mountain nyala and the current constant harvest level of 10 males ≥5 years old in a hypothetical hunting area. Density dependence in the form of reduced survival of juveniles produced a density at carrying capacity of 5075. At 7.5% poaching, the population size was stable at 889 mountain nyala, close to the estimated current population of 839, after 50 years. The population went extinct when >10% was poached (Fig.2). On this basis, we assumed mountain nyala populations can remain stable with a loss due to poaching of 7.5% and 10 males ≥5 years old taken as trophies each year. We took this as the baseline scenario that best represented the current situation.

Law enforcement through patrolling leads to 3.7 times higher mountain nyala population densities (Atickem et al. 2011). We investigated the effectiveness of the rule for adaptive harvesting under different levels of monitoring uncertainty and poaching uncertainty. To do this, we assumed companies were investing to some extent in antipoaching efforts that reduced poaching from 7.5% to 5%. We first tested the combination of a range of years of data availability (2–18 years) and flexibility in adaptive harvesting (δ = 0.0005 to δ = 0.02) in relation to the performance metrics (annual quota, average AAV, population size, percentage of times the population went extinct out of 1000 replicates). We selected the combination of years of data availability and flexibility that best balanced the different performance metrics for the next step.

Three different harvesting approaches, an adaptive-harvesting control rule (used mostly in fisheries), the proportional harvesting mostly used in hunting, and a constant harvest of 10 nyala were then evaluated for a range of monitoring uncertainties (0.01–0.2 CV) and levels of poaching (0–10% of total population) in relation to the performance metrics.

## Results

### Years of Data Availability and Flexibility in Adaptive Harvesting

**Figure 3:**
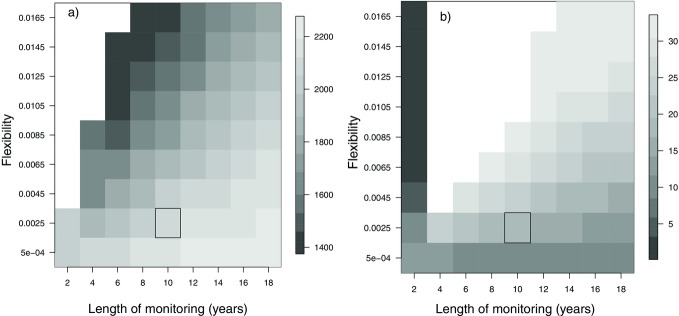
The (a) population size (extinction risk <1% in all case, white area) and (b) quota of mountain nyala (average annual variation <5 nyala per year, white area) for a range of length of monitoring years and flexibility parameters (the magnitude by which the quota changes in relation to population change). The box indicates the values chosen that balance the number of years of monitoring data used and flexibility of decision making, taking into account the size of the quota, population size, extinction risk, and annual average variation (AAV) of the quota.

There was a trade-off between the number of years of monitoring and the flexibility in quota setting when attempting to balance the competing aims of maximizing population size and quota, while keeping extinction risk and year-to-year variability of the quota below an acceptable level. More years of monitoring and lower flexibility resulted in higher population size (Fig.3a) but the lowest quota (Fig.3b). Highest quotas were reached at higher flexibility and intermediate monitoring lengths. Currently, there are 10 years of monitoring data available, which if used to set an adaptive harvest-control rule would lead to relatively high population sizes and intermediate quota sizes when coupled with low-to-intermediate flexibility (black rectangles in Figs.3a & 3b). Decreasing poaching levels from 7.5% to 5% allowed an increase in population size, an increase in quotas. With a monitoring length of 10 years, a flexibility of 0.0025, and a legal quota of 10 trophies per year, the population size and the quota more than doubled (Figs.3a & 3b).

### Constant, Proportional, and Adaptive Harvesting

**Figure 4:**
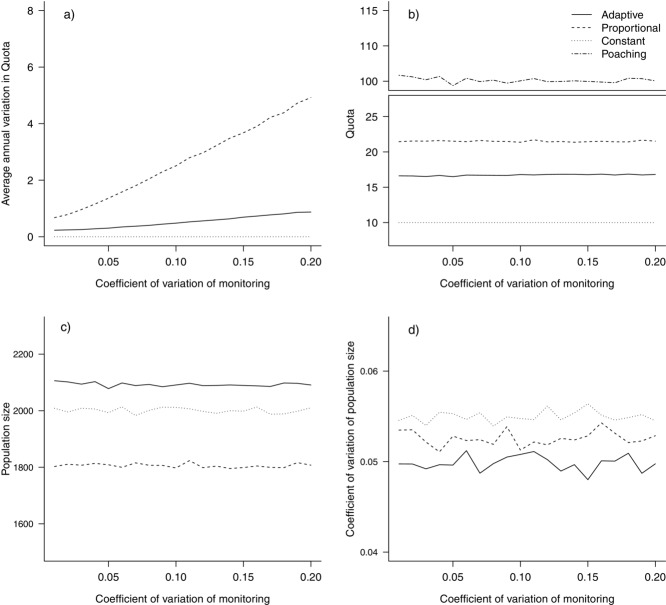
Effect of changing the coefficient of variation of monitoring from 0 to 0.2 on (a) average annual variation in quota, (b) quota size (number of nyala lost as a result of poaching included), (c) population size, and (d) coefficient of variation of population size. Results from scenarios of adaptive, proportional, and constant harvesting are shown.

An increase in the CV of monitoring led to an increase in AAV in the quota for both harvesting approaches, but at high levels (CV = 20%) of monitoring uncertainty the AAV was only 1 nyala/year for adaptive hunting compared with a variation of more than 5 in the nyala quota for proportional hunting (Fig.4a). At these high levels of uncertainty, the mean quota size was similar for both strategies (Fig.4b). However, the population was about 16% higher for the adaptive approach than for proportional harvesting (Fig.4c), and the CV of the population size was slightly lower (Fig.4d). Hence, under high monitoring uncertainty the adaptive strategy minimized annual variations in the quota while maintaining a higher population size, when compared with a more standard approach.

At a monitoring CV that is common for wildlife studies (15%), adaptive harvesting maintained a lower AAV than proportional harvesting when flexibility was low and when there were more years of available monitoring data. With more flexibility and fewer years of monitoring data, the AAV was higher for the adaptive than the proportional strategy (Supporting Information).

**Figure 5:**
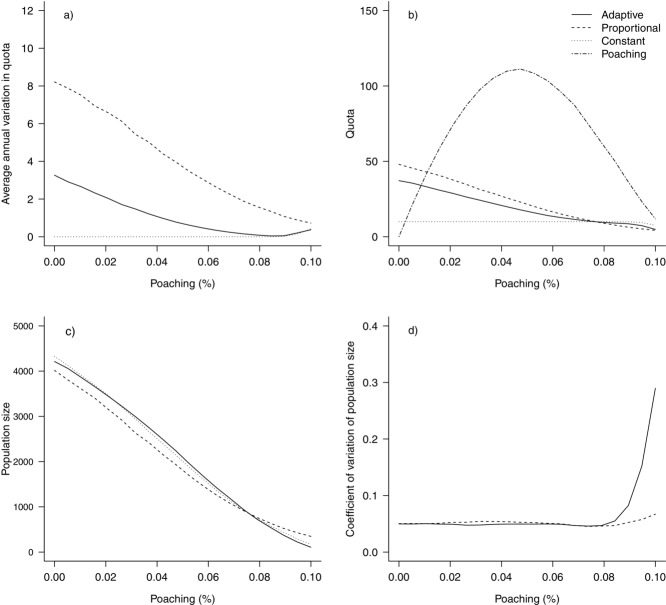
Effect of poaching of mountain nyala (percent offtake of the male population >5 years of age) on (a) average annual variation in quota, (b) quota size, (c) population size, and (d) the coefficient of variation of population size. Results from scenarios of adaptive, proportional, and constant harvesting are shown.

At low poaching levels (1%), the adaptive strategy lowered the quota AAV by up to 5 nyala/year (Fig.5a) but returned a quota size that was about 10 nyala lower compared with proportional hunting (Fig.5b). The population size was similar among the adaptive, proportional, and constant strategies (Fig.5c). At high levels of poaching (10%), the population went nearly extinct for all 3 strategies. The CV in population size was generally stable for all 3 strategies, but it increased due to low population size at high poaching rates (Fig.5d).

## Discussion

We demonstrate here the power of an MSE approach for species that are trophy hunted. The framework enabled us to consider the incentives faced by more than one stakeholder (in this case, the government and safari companies) and to compare the effects of different forms of uncertainty on the performance of harvest-control rules. The framework also allowed us to compare the effectiveness of 2 different potential management activities that improved population monitoring and reduced poaching levels.

The comparison of harvesting strategies showed that the adaptive approach outperformed proportional harvesting by reducing variation in the quota when poaching occurred at an unknown level. Using data from previous years in the decision making process (in fisheries an empirical data-driven harvest-control rule) means the adaptive approach is more likely to respond to actual population trends than to the noise inherent in annual monitoring of population abundance. The year-to-year variation in the quota is an often-overlooked measurement of paramount importance for management planning (both for safari companies and governments). A recent simulation study on salmon fisheries showed that use of multiple years of data decreased the variation in the allowable fishing opportunity with only minor effects on extinction risks (Winship et al. 2013). A constant harvest would alleviate the problem of variation but would not allow for an increase of the trophy quota (and revenues) even if the population could withstand a higher quota, for example because of better monitoring or less poaching (Figs.4b & 5b). A quota-setting rule that minimizes the quota variation (AAV) while enabling quotas to be changed where appropriate would allow managers to plan ahead and would encourage future investment in conservation and management of trophy hunting.

The flexibility parameter (which determined how the quota responded to the observed population trend) embodies important management trade-offs: variability versus stability and harvest level versus population size. Within fisheries science, flexibility parameters are tuned to the management aims being addressed. For example, the harvest rate for South African hake fishery (*Merluccuis capensis* and *M. paradoxus*) is tuned to react more quickly when the stock estimate shows a decreasing trend and less quickly when the trend is increasing or stable (Rademeyer et al. 2008). As monitoring proceeds and longer periods become available, there is more scope to alter the flexibility parameter in concert with the time window of monitoring data that contributes to quota setting to meet particular management objectives.

Ensuring that population and quota levels are maintained in the face of high levels of poaching is challenging, thus it would be in the interests of safari companies to support antipoaching activities (which is in fact what is observed) (Atickem et al. 2011). However, if the CV of monitoring >15%, this leads to potentially unacceptable interannual variation in quotas. This suggests that if the government is interested in maintaining system stability, it would be worthwhile for them to invest enough in monitoring to ensure the CV remains below 15%. And although safari companies currently do not monitor, the government does carry out a minimal level of monitoring. Results of our analyses therefore suggest one potential mechanism behind the observed behavior of 2 key stakeholders in the system and illustrate the usefulness of the MSE framework, which enables multiple viewpoints to be modeled.

The projected improvements in quota from the current level rely on poaching levels being substantially reduced (from an estimated 7.5% to 5%). Experience from the Serengeti (Hilborn et al. 2006) and from the Bale Mountains itself show that law enforcement can be effective. Patrolled hunting areas in the park had a 3.7 times higher population size than unpatrolled areas (Atickem et al. 2011). Our model shows that the population size can be up to 4 times higher in effectively patrolled areas with <1% poaching compared with areas with high poaching levels (up to 8%). Another way to reduce poaching would be to transfer benefits from trophy hunting to the local community. For example, in Tarangire National Park in Tanzania, tourism and direct payments for habitat conservation led local people to set-aside land for conservation, whereas payments, such as donations, that are not conditional on actions were not successful as a conservation tool (Sachedina & Nelson 2010). This suggests that a crucial next step in MSE development is to collect data on local people’s decision making, land-use change, and associated costs and to build these data explicitly into the modeling framework, rather than, as here, incorporating it implicitly in the form of a poaching rate (Milner-Gulland 2011).

Empirical studies have quantified the economic benefits of trophy hunting. Results of these studies suggest trophy hunting can be a valuable conservation tool (Lindsey et al. 2007). Ecological-economic models have also shed light on the decision making trade-offs local people face (e.g., between hunting and agriculture or between risk of being caught and profits from hunting) (Bulte & van Kooten 1999; Bulte & Horan 2003). A second type of ecological-economic model focuses on the decision making of a single agent balancing protection of land for conservation and use of the land for agriculture (Bulte & Horan 2003). Instead of optimizing the trade-off between hunting, agriculture, and fines, this study focused on 2 major uncertainties in the management of trophy hunting. First, information on population size is often largely uncertain due to imperfect monitoring (Butterworth & Punt 1999), especially when funding is limited, as it is in many parts of Africa (Balmford et al. 2004). Second, species that can be consumed or sold on markets often face illegal and potentially high offtakes (Milner-Gulland et al. 2003), but poaching levels are difficult to quantify (Keane et al. 2008; St John et al. 2012). Here we found that 10 years of population monitoring was sufficient to develop sustainable strategies for management even under continuing poaching. Thus, our approach contributes to the current ecological-economic literature by focusing on decision making under uncertainty, which has been identified as one of the main developments needed to advance bioeconomic modeling and management of terrestrial and marine economic-ecological systems (Bockstael et al. 1995; Watzold et al. 2006; Winship et al. 2013).

Management and conservation decisions are always made under uncertainty, and advances in conservation decision making are hampered by not fully including uncertainty in modeling studies (Langford et al. 2011). The MSE framework has the potential to incorporate the decision making processes of multiple stakeholders into modeling of terrestrial wildlife management and explicitly to include uncertainty in both monitoring and implementation of conservation interventions (Bunnefeld et al. 2011). This extends the single-objective approach (maximum sustainable yield) often used to assess the sustainability of exploitation to take into account the objectives of multiple stakeholders. Thus, our study contributes to the ongoing debate concerning the potential for sustainable management of trophy hunting as a tool for conservation of endangered species.

## Supporting Information

Female and male fecundities (Appendix S1), density dependence (Appendix S2), total population size in relation to proportional harvest rates (Appendix S3) and average annual variation in quota size between proportional and adaptive harvesting (Appendix S4) are available online. The authors are solely responsible for the content and functionality of these materials. Queries (other than absence of the material) should be directed to the corresponding author.
