# Supplementary material for: Incentivizing Monitoring and Compliance in Trophy Hunting
Source: Conserv Biol. 2014 Jan 1;27(6):1344–54. doi: 10.1111/cobi.12120 (PMC4265855; doi:10.1111/cobi.12120)
Supplement: Supplementary file 1 [file cobi0027-1344-SD1.docx]

Appendix for

**Incentivizing monitoring and compliance in trophy hunting: a Management Strategy Evaluation approach**

**Nils Bunnefeld, Charles T.T. Edwards, Anagaw Atickem, Fetene Hailu, E.J. Milner-Gulland**

Appendix1

The matrix contains both females and males because only adult males from the age of five years can be hunted for trophies. The nyala breeding system is polygynous, i.e. one male can mate with more than one female. However, the number of females a single male can mate is unknown for nyala, so we assumed the same as for greater kudu ([h=5, Caro et al. 2009](#_ENREF_10)). We expected the sex ratio to change depending on the trophy harvest rate for males, thus we calculated the contribution from males and females according to the harem size following Caswell & Weeks ([1986](#_ENREF_11)) and Lindstrom & Kokko ([1998](#_ENREF_25)). The per capita male and female fecundities depending on the sex ratio can be calculated as follows:

$F_{m,t}=\frac{0.5kN_{f,t}}{N_{m,t}+N_{f,t}h^{-1}}$ Eqn 1

$F_{f,t}=\frac{0.5kN_{m,t}}{N_{m,t}+N_{f,t}h^{-1}}$ Eqn 2

Where *F_m,t_* and *F_f,t_* is the fecundity for males and females respectively depending on the litter size *k* (times 0.5 for females producing half a litter and males the other half), the number of females *N_f_* and males *N_m_* in year *t* and the number of females a single a male can mate with *h*. Equation 2 and 3 result in an asymptotic curve with decreasing per capita female fecundity and increasing per capita male fecundity when female-to-male ratio increases (Figure below). Stochasticity was added to the survival rates and the fecundity by varying the rates by a standard deviation from a normal distribution bounded between zero and one (Table 1). We assumed an equal sex ratio at birth for all model scenarios.

Appendix 2

Density dependence was included in the model by varying juvenile survival. Using the density estimates from three hunting areas monitored by Atickem et al. ([2011](#_ENREF_2)) we determined the starting population. The population size from the two lower-density hunting areas was estimated to be 4.8 nyala km^-1^ ([Atickem et al. 2011](#_ENREF_2)) and the mean size of these hunting areas was 175 km^2^ (EWCA, pers comm) which resulted in a starting population size of 839 nyala in a hypothetical hunting area without hunting company investment in poaching reduction. The carrying capacity was calculated as the highest density that was found in a hunting area (29 nyala km^-1^) multiplied by the mean size of a hunting area (175 km^2^) resulting in a maximum population size of 5075 nyala. We used Owen-Smith’s ([2006](#_ENREF_33)) slope and intercept to calculate the juvenile survival as a function of density.

$S_{j}=1-\sigma+\frac{\varphi N_{t}}{\tau}$ Eqn 4

where *S_j_* is the survival of juveniles, *σ* the intercept, *φ* the slope, *N_t_* the total population size in year *t* and *τ* is a conversion factor because we used maximum population size rather than biomass in the original work by Owen-Smith ([2006](#_ENREF_33)).

Appendix 1 Table 1: Parameters for the mountain nyala matrix model.

| Parameter | Symbol | Parameter | Source |
| --- | --- | --- | --- |
| Fecundity standard deviation |  | 0.065 | ([Gaillard et al. 2000](#_ENREF_15)) |
| Juvenile density dependent survival (intercept) | *σ* | 0.400 | ([Owen-Smith 2006](#_ENREF_31)) |
| Juvenile density dependent survival (slope) | *φ* | 0.153 | ([Owen-Smith 2006](#_ENREF_31)) |
| Conversion factor for density dependence | *τ* | 3139 | ([Owen-Smith 2006](#_ENREF_31)) |

Appendix 3 The total population size and number of nyalas hunted for a range of proportional harvest rates. The model was run without poaching.

Appendix 4: The difference in average annual variation (AAV) in quota size between proportional and adaptive harvest strategies for a range of length of monitoring years and the flexibility parameter. A positive number indicates a lower value for the adaptive than for the proportional strategy. The coefficient of variation of monitoring is fixed to 15% and poaching rate at zero.
